# PD-L1: a novel prognostic biomarker in head and neck squamous cell carcinoma

**DOI:** 10.18632/oncotarget.17547

**Published:** 2017-05-02

**Authors:** Tim Müller, Martin Braun, Dimo Dietrich, Seher Aktekin, Simon Höft, Glen Kristiansen, Friederike Göke, Andreas Schröck, Johannes Brägelmann, Stefanie A.E. Held, Friedrich Bootz, Peter Brossart

**Affiliations:** ^1^ Institute of Pathology, University Hospital Bonn, Bonn, Germany; ^2^ Department of Otorhinolaryngology/Head and Neck Surgery, University Hospital Bonn, Bonn, Germany; ^3^ Department of Oncology, Hematology and Rheumatology, University Hospital Bonn, Bonn, Germany

**Keywords:** PD-L1, prognostic biomarker, head and neck squamous cell carcinoma, immunohistochemistry

## Abstract

**Background:**

The PD-1 receptor and its ligands PD-L1 and PD-L2 are known to be significantly involved in T-cell regulation. Recent studies suggest that PD-L1 expression in malignant tumors contributes to an immunosuppressive microenvironment and disruption of antitumoral immune response. Drugs targeting this pathway are already tested in clinical trials against several tumor entities with promising results. However, until now comprehensive data with regard to PD-L1 and PD-L2 expression in head and neck squamous cell carcinoma (HNSCC) is still lacking.

**Patients and methods:**

We assessed PD-L1 and PD-L2 expression via immunohistochemistry in two independent cohorts of 293 HNSCC patients.

**Results:**

A significant subset of HNSCC showed high expression levels of PD-L1. Most remarkable, we detected a strong correlation between PD-L1 expression and overall survival time in both HNSCC cohorts. Further, in multivariate cox proportional hazard models, PD-L1 dominates as the strongest prognostic factor of patient's outcome in HNSCC, leaving even tumor stage and distant metastasis behind. Moreover, strong PD-L1 expression was associated with the presence of distant metastases in a subset of cases.

**Conclusions:**

In summary, while the significance of PD-L2 in HNSCC seems to minor, we show that PD-L1 expression is common in HNSCC and, more importantly, a both robust and strong prognostic biomarker. In this respect, our results provide hints on further application of therapies targeting the PD-1/PD-L1 pathway in HNSCC. Investigation of response and outcome of patients receiving anti-PD-1/PD-L1 containing therapies in correlation with PD-L1 expression analysis should be an important task for the future.

**STATEMENT OF TRANSLATIONAL RELEVANCE:**

In spite of improved treatment options and increasing knowledge of molecular alterations in HNSCC, the survival rate has not been dramatically changed in the past decades. Pies are missing in HNSCC. One promising candidate in cancer immune therapy is PD-L1.

Drugs targeting PD-L1 or its receptor PD-1 are subject of several clinical studies in different cancer entities. However, comprehensive data about PD-L1 expression in HNSCC and therefore a rational basis for anti PD-L1/PD-1 therapy in HNSCC is lacking. Here, we provide wide-ranging data about PD-L1 expression in HNSCC of all major localizations. We observed a strong correlation between expression of PD-L1 and reduced overall survival time. Furthermore, high PD-L1 expression was identified as a strong prognostic factor of patient's outcome when verified together with recognized prognostic factors.

## INTRODUCTION

Head and neck squamous cell carcinoma (HNSCC), derived from squamous epithelium of the upper aerodigestive tract, is the most common malignancy in the head and neck region with over 600,000 new cases diagnosed each year. The consumption of tobacco and alcohol is strongly associated with development of HNSCC and responsible for 72% of HNSCC cases. These major risk factors act synergistically in HNSCC, but studies have demonstrated that each agent works as an independent risk factor [1–3]. Recent studies suggest human papilloma virus (HPV), in particular HPV 16, as cancer-causing agent in 22% of HNSCC cases [4]. Treatment of HNSCC is based on tumor localization and staging. Localized HNSCC can be cured by radical surgical resection. In the event of advanced HNSCC, a multidisciplinary approach, including surgical, chemotherapy and radiotherapy, is required. Unfortunately, lymph node metastasis is present in 50% of patients at the time of diagnosis and distant metastases are detected in 20-30% within the first two years after treatment of the primary tumor. Metastatic disease is associated with poor prognosis [5]. In the past decades, the survival rate has not increased remarkably. Therefore, the identification of novel biomarkers and potential molecular candidates for targeted cancer therapy is necessary. One promising candidate is the immune checkpoint regulator PD-L1 (programmed death ligand 1). PD-L1 is an immunomodulatory cell-surface glycoprotein expressed in T- and B-cells, dendritic cells, macrophages and some non-hematopoietic tissues [6]. Co-expression of PD-L1 and its receptor PD-1 (programmed death 1) causes inhibition of lymphocyte proliferation and cytokine secretion mediated by T-cell receptor [7]. On the other hand, the expression of PD-L1 is influenced by various cytokines, in which interferon-γ is playing a key role [8]. For this reason, the PD-1/PD-L1 pathway plays a crucial role in the regulation of T-cell activity during inflammatory response to infection and limitation of autoimmunity [6]. Recent studies suggest that PD-L1 expressing cancer cells have the ability to efficiently evade the host immune system. PD-L1 expression is common in many solid human cancers including urothelial cancer, colorectal cancer, gastric cancer, esophageal cancer, hepatocellular carcinoma, melanoma, glioblastoma, lung cancer, ovarian cancer, prostate cancer and oral squamous cell carcinoma [9–12]. Inhibition of the interaction between PD-1 and its ligand PD-L1 unleashes anticancer activity by enhanced T-cell response *in vitro* [13, 14]. Brahmer and colleagues demonstrated that inhibition of PD-1 using an anti-PD-1-antibody enhanced objective response rates of 6% to 17% and prolonged disease stabilization in patients with metastatic non-small-cell lung cancer, melanoma, renal-cell-carcinoma and ovarian cancer [15]. Immunohistochemical analysis of untreated tumor specimens revealed, that objective responses to anti-PD-1-therapy was higher in tumors expressing PD-L1. However, patients with PD-L1-negative tumors still showed response rates and benefit from treatment [16]. Early clinical trials in patients with recurrent or metastatic head and neck squamous cell carcinoma (HNSCC) using the anti-PD-1 antibodies nivolumab or pembrolizumab demonstrated impressive clinical activity for this patient population, which previously had limited options following progression on a platinum-based chemotherapy [17, 18]. Further, in a recently performed analysis, PD-L1 immunoreactivity was determined by immunohistochemistry in 305 cancer specimens from patients with squamous carcinomas of the oral cavity. Multivariate analysis identified high PD-L1 expression as an independent risk factor in males and smokers [10]. However, the prognostic significance in HNSCC other than from the oral cavity, and the significance of the PD-1 ligand PD-L2 in HNSCC are still unknown. We here present an extensive analysis of PD-L1 and PD-L2 expression in two well defined independent cohorts (training set and testing set) of primary HNSCC tumor samples that included tumors derived from localized or locally advanced HNSCC located in the oral cavity, oropharynx, hypopharynx and larynx. All patients included in the study were treated by surgery with optional adjuvant radio-chemotherapy or definitive radio-chemotherapy with curative intent.

## RESULTS

### PD-L1 and PD-L2 expression in HNSCC

**Figure 1:**
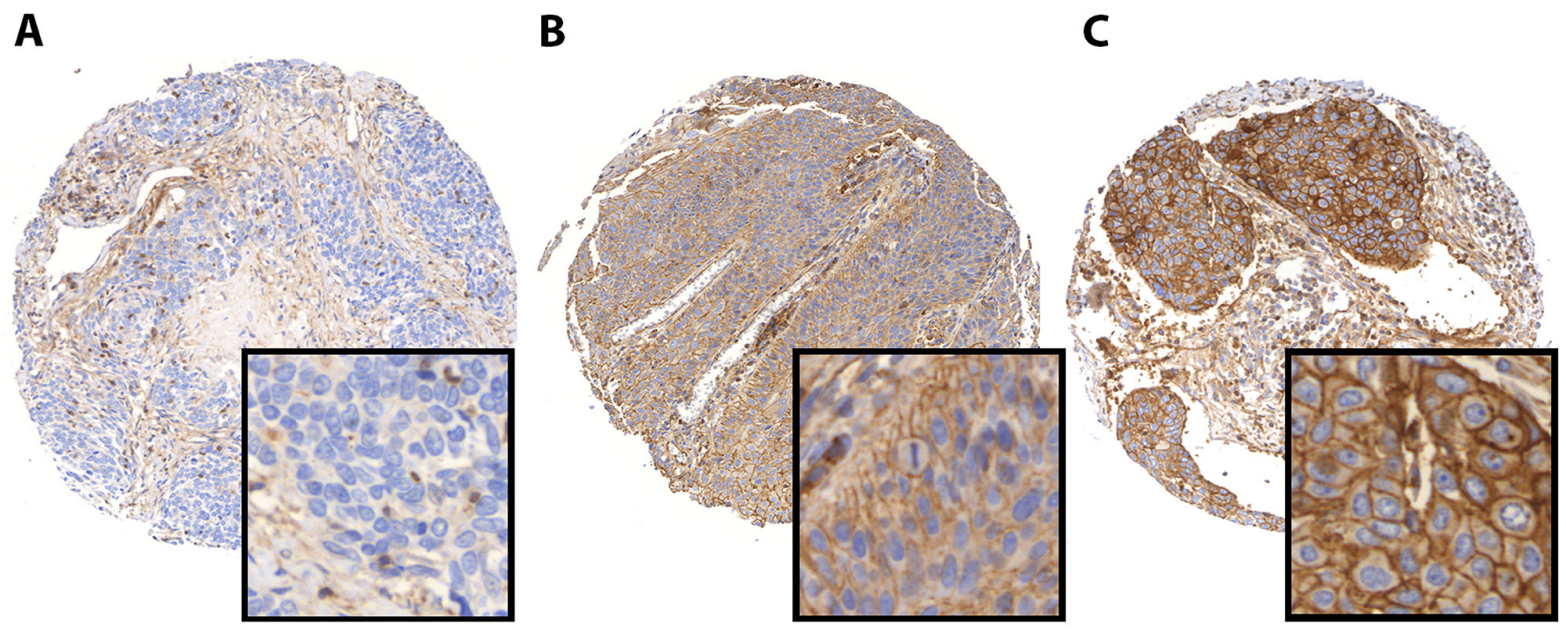
PD-L1 immunohistochemistry in HNSCC Representative images of HNSCC demonstrating negative (**A**), low (**B**), and high (**C**) PD-L1 protein levels.

PD-L1 and PD-L2 expression was mainly seen at the membrane and in the cytoplasm of the tumor cells. Interestingly, a subset of peritumoral and tumor infiltrating lymphocytes also revealed strong immunoreactivity for PD-L1 and PD-L2. Benign squamous epithelium generally showed low PD-L1 and PD-L2 expression that was mainly restricted to the lower third of the epithelium. In the first cohort, strong staining intensity for PD-L1 was seen in 15 (15%) samples, and 78 (80%) of the analyzed tumor specimen revealed low staining intensities. No immunoreactivity was observed in 5 (5%) tumor samples. In the second cohort, 54 (28%) of the investigated tumor specimen showed strong staining for PD-L1, whereas 111 (57%) samples revealed low expression levels. 30 (15%) of samples showed no tumor-specific immunoreactivity (Figure 1). For the vast majority of cases of the first cohort and for a subset of cases of the second cohort, data concerning PD-L2 expression is available.

Most tumors exhibited low staining of PD-L2, 81 (84%) tumors in the first cohort and 108 (85%) tumors in the second cohort, respectively. 2 (2%) tumor samples of the first cohort and 13 (10%) tumor specimens of the second cohort demonstrated pronounced immunoreactivity for PD-L2. No immunoreactivity was seen in 14 (14%) cases of the first cohort and 6 (5%) cases of the second cohort (Supplementary Figure 1). PD-L1 and PD-L2 were co-expressed in 80% of cases, while 20% of cases showed an inverse expression pattern. Notably, in both cohorts, no significant intratumoral heterogeneity could be detected, as the staining was highly similar in different TMA cores.

### PD-L1 and PD-L2 expression and clinico-pathological parameters

We observed no correlation between PD-L1 and PD-L2 staining intensity and well-known causative agents for HNSCC like tobacco use, alcohol consumption or presence of human papillomavirus. There was no correlation between PD-L1 with tumor stage, lymph node involvement, lymphatic invasion, vascular invasion, tumor grade or extracapsular expansion (Tables 1 and 2). Interestingly, there was an association between high PD-L2 expression and the occurrence of hemangiosis carcinomatosis in both cohorts (first cohort: p=0.030; second cohort: p=0.044). Furthermore, high tumor stage (p=0.045) as well as high tumor grade (p=0.005) were associated with higher expression levels of PD-L2 in the first cohort. However, these findings do not allow a comprehensive conclusion, particularly due to the low number of cases demonstrating high PD-L2 expression and the low number of events (Supplementary Tables 1 and 2).

**Table 1:** Clinico-pathological characteristics of 98 HNSCC included in the first cohort and association with PD-L1 expression

	Total number (n)	PD-L1 high	PD-L1 low	PD-L1 negative	*p*-value
**All HNSCC cases**	98	15 (15.31%)	78 (79.59%)	5 (5.10%)	
**Age**					***p=0.235^†^***
≤50 years	8 (8.16%)	0 (0.00%)	8 (100.00%)	0 (0.00%)	
51-60 years	25 (25.51%)	3 (12.00%)	19 (76.00%)	3 (12.00%)	
> 60 years	65 (66.33%)	12 (18.46%)	51 (78.46%)	2 (3.08%)	
Median age [years]	64				
Mean age [years]	64.27				
Age range [years]	38 – 88				
**Gender**					***p=0.883^†^***
Female	16 (16.33%)	3 (18.75%)	12 (75.00%)	1 (6.25%)	
Male	82 (83.67%)	12 (14.63%)	66 (80.49%)	4 (4.88%)	
**Follow-up**					
Mean follow-up [days]	573.93				
Median follow-up [days]	470				
Range follow-up [days]	4 – 1814				
**Tobacco use**					***p=0.484^†^***
Non-smokers	6 (6.12%)	0 (0.00%)	6 (100.00%)	0 (0.00%)	
Smokers (current and former)	40 (40.82%)	7 (17.50%)	32 (80.00%)	1 (2.50%)	
Unknown smoking status	52 (53.06%)	8 (15.38%)	40 (76.92%)	4 (7.69%)	
**Alcohol consumption**					***p=0.798^†^***
No alcohol	2 (2.04%)	1 (50.00%)	1 (50.00%)	0 (0.00%)	
Occasional	5 (5.10%)	0 (0.00%)	5 (100.00%)	0 (0.00%)	
Moderate	22 (22.45%)	2 (9.09%)	19 (86.36%)	1 (4.55%)	
Frequent	6 (6.12%)	1 (16.67%)	5 (83.33%)	0 (0.00%)	
Alcoholic (current and former)	7 (7.14%)	2 (28.57%)	5 (71.43%)	0 (0.00%)	
Unknown alcohol consumption	56 (57.14%)	9 (16.07%)	43 (76.79%)	4 (7.14%)	
**HPV status**			No data available		
**Localization**					***p=0.771^†^***
Oral cavity	8 (8.16%)	1 (12.50%)	7 (87.50%)	0 (0.00%)	
Oropharnyx	58 (59.18%)	8 (13.79%)	46 (79.31%)	4 (6.90%)	
hypopharnyx	5 (5.10%)	0 (0.00%)	5 (100.00%)	0 (0.00%)	
larynx	27 (27.55%)	6 (22.22%)	20 (74.07%)	1 (3.70%)	
**T-stage**					***p=0.581^†^***
Tis	1 (1.02%)	0 (0.00%)	1 (100.00%)	0 (0.00%)	
T1	19 (19.39%)	2 (10.53%)	17 (89.47%)	0 (0.00%)	
T2	37 (37.76%)	5 (13.51%)	30 (81.08%)	2 (5.41%)	
T3	28 (28.57%)	5 (17.86%)	22 (78.57%)	1 (3.57%)	
T4	12 (12.24%)	3 (25.00%)	7 (58.33%)	2 (16.67%)	
Tx	1 (1.02%)	0 (0.00%)	1 (100.00%)	0 (0.00%)	
**Lymph node involvement**					***p=0.758^†^***
N0	37 (37.76%)	4 (10.81%)	31 (83.78%)	2 (5.41%)	
N1	13 (13.27%)	4 (30.77%)	9 (69.23%)	0 (0.00%)	
N2	39 (39.80%)	7 (17.95%)	29 (74.36%)	3 (7.69%)	
N3	2 (2.04%)	0 (0.00%)	2 (100.00%)	0 (0.00%)	
Nx	7 (7.14%)	0 (0.00%)	7 (100.00%)	0 (0.00%)	
**Distant metastases**					***p=0.864^†^***
M0	66 (67.35%)	11 (16.67%)	51 (77.27%)	4 (6.06%)	
M1	1 (1.02%)	0 (0.00%)	1 (100.00%)	0 (0.00%)	
n.a.	31 (31.63%)	4 (12.9%)	26 (83.87%)	1 (3.23%)	
**Grading**					***p=0.427^†^***
G1	2 (2.04%)	0 (0.00%)	2 (100.00%)	0 (0.00%)	
G2	53 (54.08%)	6 (11.32%)	45 (84.91%)	2 (3.77%)	
G3	39 (39.80%)	9 (23.08%)	27 (69.23%)	3 (7.69%)	
n/a	4 (4.08%)	0 (0.00%)	4 (100.00%)	0 (0.00%)	
**Lymphatic invasion**					***p=0.454^†^***
L0	67 (68.37%)	10 (14.93%)	55 (82.09%)	2 (2.99%)	
L1	21 (21.43%)	3 (14.29%)	16 (76.19%)	2 (9.53%)	
n/a	10 (10.20%)	2 (20.00%)	7 (70.00%)	1 (10.00%)	
**Vascular invasion**					***p=0.433^†^***
V0	75 (76.53%)	10 (13.33%)	63 (84.00%)	2 (2.76%)	
V1	11 (11.22%)	3 (27.27%)	8 (72.73%)	0 (0.00%)	
n/a	12 (12.24%)	2 (16.67%)	7 (58.33%)	3 (25.00%)	
**Extracapsular expansion**					***p=0.877^†^***
ece-	21 (21.43%)	4 (19.05%)	16 (76.19%)	1 (4.76%)	
ece+	28 (28.57%)	4 (14.29%)	23 (82.14%)	1 (3.57%)	
n/a	49 (50.00%)	7 (14.29%)	39 (79.59%)	3 (6.12%)	
**Surgical margin**					***p=0.411^†^***
R0	69 (70.41%)	8 (11.59%)	56 (81.16%)	5 (7.25%)	
R1	19 (19.39%)	5 (26.32%)	14 (73.68%)	0 (0.00%)	
R2	1 (1.02%)	0 (0.00%)	1 (100.00%)	0 (0.00%)	
n/a	9 (9.18%)	2 (22.22%)	7 (77.78%)	0 (0.00%)	

^†^X^2^-test (Pearson)

**Table 2:** Clinico-pathological characteristics of 195 HNSCC included in the second cohort and association with PD-L1 expression

	Total number (n)	PD-L1 high	PD-L1 low	PD-L1 negative	*p*-value
**All HNSCC cases**	195 (100%)	54 (27.69%)	111 (56.92%)	30 (15.38%)	
**Age**					***p=0.044^†^***
≤50 years	29 (14.87%)	13 (44.83%)	15 (51.72%)	1 (3.45%)	
51-60 years	50 (25.64%)	16 (32.00%)	24 (48.00%)	10 (20.00%)	
> 60 years	116 (59.49%)	25 (21.55%)	72 (62.07%)	19 (16.38%)	
Median age [years]	62				
Mean age [years]	62.41				
Age range [years]	27-87				
**Gender**					***p=0.673^†^***
Female	53 (27.18%)	17 (32.08%)	29 (54.72%)	7 (13.21%)	
Male	142 (72.82%)	37 (26.06%)	82 (57.75%)	23 (16.20%)	
**Follow-up**					
Mean follow-up [days]	856				
Median follow-up [days]	791				
Range follow-up [days]	1-2566				
**Tobacco use**					***p=0.798^†^***
Non-smokers	23 (11.79%)	7 (30.43%)	12 (52.17%)	4 (17.39%)	
Smokers (current and former)	114 (58.46%)	30 (26.32%)	68 (59.65%)	16 (14.04%)	
Unknown smoking status	58 (29.74%)	17 (29.31%)	31 (53.45%)	10 (17.24%)	
**Alcohol consumption**					***p=0.147^†^***
No alcohol	51 (26.15%)	17 (33.33%)	30 (58.82%)	4 (7.84%)	
Ocasional alcohol	28 (14.36%)	5 (17.86%)	19 (67.86%)	4 (14.29%)	
Alcoholic (current and former)	51 (26.15%)	12 (23.53%)	27 (52.94%)	12 (23.53%)	
Unknown alcohol consumption	65 (33.33%)	20 (30.77%)	35 (53.85%)	10 (15.38%)	
**HPV status**					***p=0.916^†^***
Negative	179 (91.79%)	50 (27.93%)	102 (56.98%)	27 (15.08%)	
Positive	16 (8.21%)	4 (25.00%)	9 (56.25%)	3 (18.75%)	
**Localization**					***p=0.026^†^***
Oral cavity	54 (27.69%)	17 (31.48%)	27 (50.00%)	10 (18.52%)	
Oropharnyx	79 (40.51%)	20 (25.32%)	46 (58.23%)	13 (16.46%)	
hypopharnyx	18 (9.23%)	8 (44.44%)	5 (27.78%)	5 (27.78%)	
larynx	44 (22.56%)	9 (20.45%)	33 (75.00%)	2 (4.55%)	
**T-stage**					***p=0.138^†^***
T1	54 (27.69%)	8 (14.81%)	36 (66.67%)	10 (18.52%)	
T2	64 (32.82%)	20 (31.25%)	34 (53.13%)	10 (15.63%)	
T3	48 (24.62%)	13 (27.08%)	30 (62.5%)	5 (10.42%)	
T4	25 (12.82%)	11 (44.00%)	10 (40%)	4 (16%)	
Tx	4 (2.05%)	2 (50.00%)	1 (25.00%)	1 (25.00%)	
**Lymph node involvement**					***p=0.115^†^***
N0	86 (44.10%)	19 (22.09%)	54 (62.79%)	13 (15.12%)	
N1	29 (14.87%)	6 (20.69%)	20 (68.97%)	3 (10.34%)	
N2	69 (35.38%)	27 (39.13%)	29 (42.03%)	13 (18.84%)	
N3	2 (1.03%)	1 (50.00%)	1 (50.00%)	0 (0.00%)	
Nx	9 (4.62%)	1 (11.11%)	7 (77.78%)	1 (11.11%)	
**Distant metastases**					***p=0.025^†^***
M0	188 (96.41%)	49 (26.06%)	110 (58.51%)	29 (15.43%)	
M1	7 (3.59%)	5 (71.43%)	1 (14.29%)	1 (14.29%)	
**Grading**					***p=0.697^†^***
G1	7 (3.59%)	1 (14.29%)	4 (57.14%)	2 (28.57%)	
G2	107 (54.87%)	29 (27.10%)	63 (58.88%)	15 (14.02%)	
G3	45 (23.08%)	15 (33.33%)	23 (51.11%)	7 (15.56%)	
n/a	36 (18.46%)	9 (25.00%)	21 (58.33%)	6 (16.67%)	
**Lymphatic invasion**					***p=0.465^†^***
L0	88 (45.13%)	24 (27.27%)	46 (52.27%)	18 (20.45%)	
L1	22 (11.28%)	7 (31.82%)	13 (59.09%)	2 (9.09%)	
n/a	85 (43.59%)	23 (27.06%)	52 (61.18%)	10 (11.76%)	
**Vascular invasion**					***p=0.388^†^***
V0	96 (49.23%)	26 (27.08%)	51 (53.13%)	19 (19.79%)	
V1	8 (4.10%)	4 (50.00%)	3 (37.50%)	1 (12.50%)	
n/a	91 (46.67%)	24 (26.37%)	57 (62.64%)	10 (10.99%)	
**Extracapsular expansion**					***p=0.235^†^***
ece-	73 (37.44%)	21 (28.77%)	40 (54.79%)	12 (16.44%)	
ece+	28 (14.36%)	13 (46.43%)	11 (39.29%)	4 (14.29%)	
n/a	94 (48.21%)	20 (21.28%)	60 (63.83%)	14 (14.89%)	
**Surgical margin**					***p=0.942^†^***
R0	136 (69.74%)	41 (30.15%)	74 (54.41%)	21 (15.44%)	
R1	10 (5.13%)	3 (30.00%)	6 (60.00%)	1 (10.00%)	
R2	3 (1.54%)	1 (33.33%)	2 (66.67%)	0 (0.00%)	
n/a	46 (23.59%)	9 (19.57%)	29 (63.04%)	8 (17.39%)	

^†^X^2^-test (Pearson)

### PD-L1 and PD-L2 expression and survival analyses

**Figure 2:**
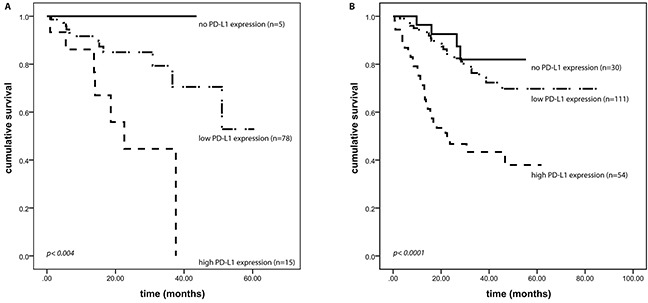
PD-L1 expression and outcome PD-L1 expression correlates with overall survival of HNSCC patients. All patients had a localized or locally advanced disease and were treated with curative intent. (**A**) first HNSCC cohort (p<0.004), (**B**) second HNSCC cohort (p<0.0001).

We found a strong correlation between PD-L1 expression and overall survival in both the first (p<0.004, Figure 2) and the second (p<0.0001, Figure 2) HNSCC cohort. Mean survival time in HNSCC showing low and high PD-L1 expression was 1452 days vs. 735 days in the first cohort, and 2045 days vs. 989 days in the second cohort, respectively. In both cohorts staining intensity of PD-L1 proved to be a prognostic factor for overall survival time in univariate proportional hazards model analysis (first cohort: *p=0.002*, HR=4.269 [95%Cl=1.733 – 10.514]; second cohort: p<0.0001, HR=2.845 [95%CI=1.808 – 4.479]).

In a multivariate cox proportional hazards model, high expression of PD-L1 prevailed to be an independent and in fact the strongest prognostic factor of patient's outcome, even when verified together with recognized prognostic factors like tumor size, lymph node involvement, distant metastases, surgical margin status, lymphatic invasion, vascular invasion, grading, and extracapsular expansion (*p=0.02*; HR=2.926 [95%CI=1.183 – 7.235]; Table 3). Moreover, in primary HNSCC, strong PD-L1 expression was associated with the occurrence of distant metastases in the further clinical course (p<0.03) in the second cohort. This association could not be found in the first cohort. We conducted the same analyses for PD-L2, and found no correlation between PD-L2 expression intensity and overall survival using Kaplan-Meier survival analysis as well as univariate and multivariate cox regression models in both cohorts (data not shown).

**Table 3:** Univariate and multivariate Cox analyses on overall survival in the first (n=98) and second HNSCC cohort (n=195) either treated by surgery with optional adjuvant radio-chemotherapy or definitive radio-chemotherapy. All patients had a localized or locally advanced disease and were treated with curative intent

	Univariate Cox analysis				Multivariate Cox analysis	
	First HNSSC cohort (n=98)		Second HNSCC cohort (n=195)		All patients	
	Hazard ratio [95% CI]	p-value	Hazard ratio [95% CI]	p-value	Hazard ratio [95% CI]	p-value
**Tumor stage** (pT_4_ vs pT_3_ vs pT_2_ vs pT_1_ vs pT_is_)	1.879 [1.155 – 3.058]	*0.010*	1.620 [1.241 – 2.114]	*0.000*	1.174 [0.786 - 1.755]	*0.433*
**Nodal Stage** (pN1 vs pN2 vs pN3 vs pN0)	1.576 [0.955 – 2.604]	*0.070*	2.272 [1.263 – 4.088]	*0.004*	4.266 [0.531 - 34.282]	*0.172*
**Distant metastases** (M_1_ vs M_0_)	0.049 [0.000 – 5.545]	*0.660*	3.661 [1.452 – 9.231]	*0.020*	0.877 [0.172 - 4.474]	*0.874*
**Grade** (G_3_ vs G_2_ vs G_1_)	2.440 [0.972 – 6.127]	*0.049*	1.115 [0.639 – 1.946]	*0.702*	1.015 [0.433 - 2.381]	*0.972*
**Lymphatic invasion** (L_1_ vs L_0_)	2.503 [0.961 – 6.523]	*0.067*	2.363 [1.079 – 5.175]	*0.044*	1.358 [0.590 - 3.125]	*0.472*
**Vascular Invasion** (V_1_ vs V_0_)	3.080 [0.988 – 9.605]	*0.080*	5.298 [1.925 – 14.577]	*0.006*	1.070 [0.316 - 3.619]	*0.914*
**Extracapsular expansion** (ece+ vs ece-)	5.822 [0.726 – 46.681]	*0.041*	1.698 [0.834 – 3.453]	*0.160*	0.931 [0.376 - 2.306]	*0.877*
**Surgical margin** (R_1_ vs R_0_)	6.129 [2.767 – 13.576]	*0,000*	1.664 [0.912 – 3.038]	*0.136*	1.411 [0.684 - 2.907]	*0.351*
**PD-L1 expression** (high vs low vs none)	4.269 [1.733 – 10.514]	*0.002*	2.845 [1.808 – 4.479]	*0.000*	2.926 [1.183 - 7.235]	*0.020*

## DISCUSSION

PD-L1 overexpression was associated with worse overall survival in several human cancers including esophageal cancer, gastric cancer, hepatocellular carcinoma, urothelial cancer, colorectal cancer and oropharyngeal squamous cell carcinoma [10–12]. Generally, it is assumed that PD-L1 enhances an aggressive tumor phenotype by allowing tumor cells to evade the host immune system by establishing an immunosuppressive microenvironment [8]. Several monoclonal antibodies targeting PD-1 or its ligand PD-L1 have been developed and are included in various clinical trials with promising results [19–24]. Most remarkably, Topalian and colleagues reported that objective response to anti PD-1/PD-L1 targeted therapy was only seen in PD-L1 positive tumors [16]. Several clinical trials are ongoing, testing the use of drugs blocking the PD-1/PD-L1 axis in HNSCC [17–18]. While some data is already available for HNSCC of the oral cavity [10, 11, 25, 26, 27], comprehensive data concerning PD-L1 expression in HNSCC is still lacking. This induced us to analyze the expression profile of PD-L1 in two large cohorts of primary HNSCC including tumors of the oral cavity, oropharynx, hypopharynx and larynx. In line with former studies examining PD-L1 expression in various cancers [10–12], we found that high PD-L1 expression is also common in HNSCC and associated with poor outcome, independent of tumor origin. Furthermore, using multivariate analysis, we verified PD-L1 expression as a prognostic biomarker, independently of other well-known prognostic factors such as tumor stage and tumor grade. Remarkably, in both the univariate and multivariate analysis, PD-L1 expression was a strong predictor for poor outcome, even leaving tumor stage and distant metastasis behind. We did also demonstrate an association between distant metastases and high PD-L1 expression in the primary tumor, suggesting that metastases might be supported by a disrupted antitumor immune response [28]. This was also observed by the two studies analyzing HNSCC of the oral cavity [10, 25]. Lin et al., who investigated the influence of PD-L1 expression on oral HNSCC in an Asian cohort, further described a correlation between high PD-L1 expression in smokers and overall survival time [10]. In the present study, however, we found that the prognostic effect of PDL1 expression seems to be independent of tobacco use. Interestingly, in a minor subset of oral HNSCC, *PD-L1* amplifications seem to be responsible for PD-L1 overexpression, as demonstrated by Straub et al. In spite of that, *PD-L1* amplification was not associated with unfavorable prognostic factors or outcome [11]. For the most part, the mechanisms leading to PD-L1 overexpression are still not fully understood, as is the fact, whether PD-L1 expression is truly a rational basis for anti PD-L1/PD-1 therapy. In contrast to our findings regarding PD-L1, PD-L2 was predominantly expressed at weak levels in HNSCC. Even though PD-L1 and PD-L2 were co-expressed in 80% of HNSCC, PD-L2 expression was not associated with survival. In contrast, we found an association of high PD-L2 expression and hemangiosis carcinomatosa as well as advanced tumor stage. However, due to the relatively low number of HNSCC highly expressing PD-L2 (cohort 1: n=2, cohort 2: n=13), these findings need to be validated before drawing comprehensive conclusions.

It is worth mentioning that as of today, there is no gold standard established for measuring PD-L1 expression in formalin fixed, paraffin embedded (FFPE) specimens. Additionally, most clinical trials investigating anti-PD-1/PD-L1 drugs use proprietary antibodies. Recently, we demonstrated that the utilization of the novel monoclonal rabbit antibody against PD-L1 (clone EPR1161) on FFPE tissue is specific and independent of the used detection system [9]. Notably, the same antibody has been successfully used by several other groups who recently assessed PD-L1 expression in various cancer entities [29–31]. In the present study, the immunoreactivity in HNSCC showed little intratumoral heterogeneity.

In summary, we are the first to demonstrate the prognostic value of PD-L1 expression in HNSCC of all major localizations. On the basis of our results, it seems likely, that HNSCC with a high PD-L1 expression and putative activation of the PD-1/PD-L1 pathway represent a highly aggressive cancer phenotype, independent of tumor origin, tumor stage or grade. Perhaps, treatment-related acute and late toxicity of conventional radiochemotherapy could be reduced by complementary therapies targeting the PD-1/PD-L1 pathway in the future [17, 18, 32]. Lastly, our results indicate that even small and localized HNSCC with high PD-L1 expression potentially follow an unfavorable clinical course, and thus might benefit from early treatment with check-point inhibitors and provide the rationale for the use of these approaches in the adjuvant setting. Interestingly, based on the current results in the metastatic disease therapy with check point inhibitors this patient population seems to benefit more from immunotherapeutic approaches and thus might have a better long term survival.

## MATERIALS AND METHODS

### Patient population and tumor specimens

This study includes two independent cohorts of paraffin-embedded primary HNSCC specimens, which were obtained from the archive of the Institute of Pathology, University of Bonn Medical Center (Bonn, Germany). Two pathologists histologically confirmed the tumor diagnosis.

The first HNSCC cohort (training set) consists of 98 patients, in detail 16 female (16%) and 82 male (84%) patients. The median age was 64 years, ranging from 38 to 88 years. In the second cohort (testing set), 53 female (27%) and 142 male (73%) patients were included, the age distribution ranged from 27 to 82 years. The median age was 62 years. Both cohorts included tumors derived from the oral cavity, oropharynx, hypopharynx and larynx. Patients included in the study had a localized or locally advanced disease. All patients were treated by surgery with optional adjuvant radio-chemotherapy or definitive radio-chemotherapy with curative intent. All investigations were conducted in an anonymous manner, as approved by the local ethics committee at the University Hospital of Bonn (Bonn, Germany).

### Tissue microarray (TMA) construction

TMAs were constructed as previously described [33]. Briefly, one to five cores with a diameter of 1.2 mm represented each case in the first HNSCC cohort. In the second HNSCC cohort, each case was represented by one to three tissue cores with a core diameter of 0.6 mm. Of note, the vast majority of TMA cores included the invasion front of the tumor.

### Immunohistochemistry

Paraffin-embedded tissue samples were cut in sections of 3 μm and mounted on Super Frost Plus slides (Thermo Fisher, Waltham, MA, USA). Slides were routinely processed, deparaffinized in xylene and rehydrated in a degraded alcohol sequence. Immunohistochemistry was performed on Ventana BenchMark Ultra automated staining system (Ventana, Tucson, AZ, USA) and visualized with the Ventana amplifier detection kit using PD-L1, clone EPR1161 (2) antibody (Abcam, Cambridge, UK, dilution 1:75) and PD-L2, clone 176611 antibody (R&D systems, Minneapolis, USA, dilution 1:600) Slides were counterstained with hematoxylin. Following dehydration in a graded alcohol sequence and steadied in pertex® (Medite GmbH, Burgdorf, Germany). Validation of the specificity of this PD-L1 antibody was performed previously [9].

### Evaluation of immunoreactivity

Immunohistochemical stainings were examined independently by two pathologists (M.B., T.M.), who were blinded to patients' clinical outcome. Membranous and cytoplasmic staining of tumor cells was considered. The first HNSCC cohort served as a training set for identifying suitable PD-L1 and PD-L2 expression thresholds for subsequent statistical analyses. As a result, PD-L1 and PD-L2 expression was scored semiquantitatively as negative (0), low (1) or strong (2) in both HNSCC cohorts. In detail, cases with no expression were scored “negative”, specimen with only minor intensity were scored “low”, and cases with a strong expression were scored “high”. To our experience, cytoplasmic PD-L1 expression was only detected in combination with a membranous staining in the same tumor cell. Vice versa, there were tumor cells which only displayed a membranous staining, and no cytoplasmic PD-L1 expression. We did not observe tumor cells with solitary cytoplasmic PD-L1 expression. We previously validated the specificity of the PD-L1 antibody in a preceding study [9].

### Statistical analysis

Statistical analyses were carried out using the SPSS 22 software package (IBM, Armonk, NY). PD-L1 protein expression was correlated with clinical-pathological data available using Chi-Square test or Pearson correlation. Overall survival time was defined as the time between first diagnosis and death of the patient or last clinical follow-up if the patient was still alive, respectively. Survival time was estimated by Kaplan-Meier analyses and compared among patient subsets using log-rank tests. Protein expression was also examined within univariate and multivariate Cox proportional hazards regression models. All statistical tests were two-sided. P-values <0.05 were considered to be statistically significant.

## SUPPLEMENTARY FIGURE AND TABLES







## References

[1] Ogden GR (2005). Alcohol and oral cancer. Alcohol.

[2] Hashibe M, Brennan P, Benhamou S, Castellsague X, Chen C, Curado MP, Dal Maso L, Daudt AW, Fabianova E, Fernandez L, Wunsch-Filho V, Franceschi S, Hayes RB (2007). Alcohol drinking in never users of tobacco, cigarette smoking in never drinkers, and the risk of head and neck cancer: pooled analysis in the International Head and Neck Cancer Epidemiology Consortium. J Natl Cancer Inst.

[3] Hashibe M, Brennan P, Chuang SC, Boccia S, Castellsague X, Chen C, Curado MP, Dal Maso L, Daudt AW, Fabianova E, Fernandez L, Wunsch-Filho V, Franceschi S (2009). Interaction between tobacco and alcohol use and the risk of head and neck cancer: pooled analysis in the International Head and Neck Cancer Epidemiology Consortium. Cancer Epidemiol Biomarkers Prev.

[4] Dayyani F, Etzel CJ, Liu M, Ho CH, Lippman SM, Tsao AS (2010). Meta-analysis of the impact of human papillomavirus (HPV) on cancer risk and overall survival in head and neck squamous cell carcinomas (HNSCC). Head Neck Oncol.

[5] Denaro N, Russi EG, Adamo V, Merlano MC (2014). State-of-the-art and emerging treatment options in the management of head and neck cancer: news from 2013. Oncology.

[6] Keir ME, Butte MJ, Freeman GJ, Sharpe AH (2008). PD-1 and its ligands in tolerance and immunity. Annu Rev Immunol.

[7] Freeman GJ, Long AJ, Iwai Y, Bourque K, Chernova T, Nishimura H, Fitz LJ, Malenkovich N, Okazaki T, Byrne MC, Horton HF, Fouser L, Carter L (2000). Engagement of the PD-1 immunoinhibitory receptor by a novel B7 family member leads to negative regulation of lymphocyte activation. J Exp Med.

[8] Zou W, Chen L (2008). Inhibitory B7-family molecules in the tumour microenvironment. Nat Rev Immunol.

[9] Gevensleben H, Dietrich D, Golletz C, Steiner S, Jung M, Thiesler T, Majores M, Stein J, Uhl B, Muller S, Ellinger J, Stephan C, Jung K (2016). The immune checkpoint regulator PD-L1 is highly expressed in aggressive primary prostate cancer. Clin Cancer Res.

[10] Lin YM, Sung WW, Hsieh MJ, Tsai SC, Lai HW, Yang SM, Shen KH, Chen MK, Lee H, Yeh KT, Chen CJ (2015). High PD-L1 expression correlates with metastasis and poor prognosis in oral squamous cell carcinoma. PLoS One.

[11] Straub M, Drecoll E, Pfarr N, Weichert W, Langer R, Hapfelmeier A, Gotz C, Wolff KD, Kolk A, Specht K (2016). CD274/PD-L1 gene amplification and PD-L1 protein expression are common events in squamous cell carcinoma of the oral cavity. Oncotarget.

[12] Wu P, Wu D, Li L, Chai Y, Huang J (2015). PD-L1 and survival in solid tumors: a meta-analysis. PLoS One.

[13] Dong H, Strome SE, Salomao DR, Tamura H, Hirano F, Flies DB, Roche PC, Lu J, Zhu G, Tamada K, Lennon VA, Celis E, Chen L (2002). Tumor-associated B7-H1 promotes T-cell apoptosis: a potential mechanism of immune evasion. Nat Med.

[14] Iwai Y, Ishida M, Tanaka Y, Okazaki T, Honjo T, Minato N (2002). Involvement of PD-L1 on tumor cells in the escape from host immune system and tumor immunotherapy by PD-L1 blockade. Proc Natl Acad Sci U S A.

[15] Brahmer JR, Tykodi SS, Chow LQ, Hwu WJ, Topalian SL, Hwu P, Drake CG, Camacho LH, Kauh J, Odunsi K, Pitot HC, Hamid O, Bhatia S (2012). Safety and activity of anti-PD-L1 antibody in patients with advanced cancer. N Engl J Med.

[16] Topalian SL, Hodi FS, Brahmer JR, Gettinger SN, Smith DC, McDermott DF, Powderly JD, Carvajal RD, Sosman JA, Atkins MB, Leming PD, Spigel DR, Antonia SJ (2012). Safety, activity, and immune correlates of anti-PD-1 antibody in cancer. N Engl J Med.

[17] (2016). Nivolumab doubles survival for patients with HNSCC. Cancer Discov.

[18] Seiwert TY, Burtness B, Mehra R, Weiss J, Berger R, Eder JP, Heath K, McClanahan T, Lunceford J, Gause C, Cheng JD, Chow LQ (2016). Safety and clinical activity of pembrolizumab for treatment of recurrent or metastatic squamous cell carcinoma of the head and neck (KEYNOTE-012): an open-label, multicentre, phase 1b trial. Lancet Oncol.

[19] Berger R, Rotem-Yehudar R, Slama G, Landes S, Kneller A, Leiba M, Koren-Michowitz M, Shimoni A, Nagler A (2008). Phase I safety and pharmacokinetic study of CT-011, a humanized antibody interacting with PD-1, in patients with advanced hematologic malignancies. Clin Cancer Res.

[20] Garon EB, Rizvi NA, Hui R, Leighl N, Balmanoukian AS, Eder JP, Patnaik A, Aggarwal C, Gubens M, Horn L, Carcereny E, Ahn MJ, Felip E (2015). Pembrolizumab for the treatment of non-small-cell lung cancer. N Engl J Med.

[21] Hamid O, Robert C, Daud A, Hodi FS, Hwu WJ, Kefford R, Wolchok JD, Hersey P, Joseph RW, Weber JS, Dronca R, Gangadhar TC, Patnaik A (2013). Safety and tumor responses with lambrolizumab (anti-PD-1) in melanoma. N Engl J Med.

[22] Robert C, Long GV, Brady B, Dutriaux C, Maio M, Mortier L, Hassel JC, Rutkowski P, McNeil C, Kalinka-Warzocha E, Savage KJ, Hernberg MM, Lebbe C (2015). Nivolumab in previously untreated melanoma without BRAF mutation. N Engl J Med.

[23] Westin JR, Chu F, Zhang M, Fayad LE, Kwak LW, Fowler N, Romaguera J, Hagemeister F, Fanale M, Samaniego F, Feng L, Baladandayuthapani V, Wang Z (2014). Safety and activity of PD1 blockade by pidilizumab in combination with rituximab in patients with relapsed follicular lymphoma: a single group, open-label, phase 2 trial. Lancet Oncol.

[24] Brahmer J, Reckamp KL, Baas P, Crino L, Eberhardt WE, Poddubskaya E, Antonia S, Pluzanski A, Vokes EE, Holgado E, Waterhouse D, Ready N, Gainor J (2015). Nivolumab versus docetaxel in advanced squamous-cell non-small-cell lung cancer. N Engl J Med.

[25] Cho YA, Yoon HJ, Lee JI, Hong SP, Hong SD (2011). Relationship between the expressions of PD-L1 and tumor-infiltrating lymphocytes in oral squamous cell carcinoma. Oral Oncol.

[26] Msaouel P, Massarelli E (2016). Immune checkpoint therapy in head and neck cancers. Cancer J.

[27] Ukpo OC, Thorstad WL, Lewis JS (2013). B7-H1 expression model for immune evasion in human papillomavirus-related oropharyngeal squamous cell carcinoma. Head Neck Pathol.

[28] Pardoll DM (2012). The blockade of immune checkpoints in cancer immunotherapy. Nat Rev Cancer.

[29] Darb-Esfahani S, Kunze CA, Kulbe H, Sehouli J, Wienert S, Lindner J, Budczies J, Bockmayr M, Dietel M, Denkert C, Braicu I, Johrens K (2016). Prognostic impact of programmed cell death-1 (PD-1) and PD-ligand 1 (PD-L1) expression in cancer cells and tumor-infiltrating lymphocytes in ovarian high grade serous carcinoma. Oncotarget.

[30] Kiyasu J, Miyoshi H, Hirata A, Arakawa F, Ichikawa A, Niino D, Sugita Y, Yufu Y, Choi I, Abe Y, Uike N, Nagafuji K, Okamura T (2015). Expression of programmed cell death ligand 1 is associated with poor overall survival in patients with diffuse large B-cell lymphoma. Blood.

[31] Nduom EK, Wei J, Yaghi NK, Huang N, Kong LY, Gabrusiewicz K, Ling X, Zhou S, Ivan C, Chen JQ, Burks JK, Fuller GN, Calin GA (2016). PD-L1 expression and prognostic impact in glioblastoma. Neuro Oncol.

[32] Merlano MC, Monteverde M, Colantonio I, Denaro N, Lo Nigro C, Natoli G, Giurlanda F, Numico G, Russi E (2012). Impact of age on acute toxicity induced by bio- or chemo-radiotherapy in patients with head and neck cancer. Oral Oncol.

[33] Goke F, Bode M, Franzen A, Kirsten R, Goltz D, Goke A, Sharma R, Boehm D, Vogel W, Wagner P, Lengerke C, Kristiansen G, Kirfel J (2013). Fibroblast growth factor receptor 1 amplification is a common event in squamous cell carcinoma of the head and neck. Mod Pathol.

