# Supplementary material for: PD-L1: a novel prognostic biomarker in head and neck squamous cell carcinoma
Source: Oncotarget. 2017 May 2;8(32):52889–900. doi: 10.18632/oncotarget.17547 (PMC5581079; doi:10.18632/oncotarget.17547)
Supplement: Supplementary file 1 [file oncotarget-08-52889-s001.pdf]

PD-L1: a novel prognostic biomarker in head and neck squamous cell carcinoma

SUPPLEMENTARY MATERIALS

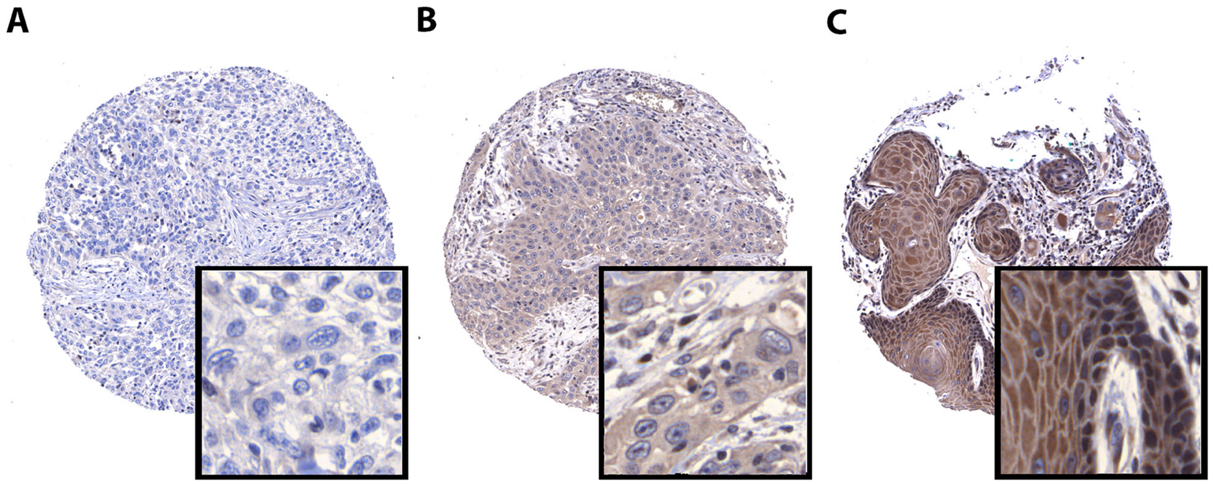

Supplementary Figure 1: PD-L2 immunohistochemistry in HNSCC. Representative images of HNSCC demonstrating negative **(A)**, low **(B)**, and high **(C)** PD-L2 protein levels.

Supplementary Table 1: Clinico-pathological characteristics of 97 HNSCC of the first cohort and association with PD-L2 expression.

See Supplementary File 1

Supplementary Table 2: Clinico-pathological characteristics of 127 HNSCC of the second cohort and association with PD-L2 expression.

See Supplementary File 2
