# Supplementary material for: PD-L1: a novel prognostic biomarker in head and neck squamous cell carcinoma
Source: Oncotarget. 2017 May 2;8(32):52889–900. doi: 10.18632/oncotarget.17547 (PMC5581079; doi:10.18632/oncotarget.17547)
Supplement: Supplementary file 2 [file oncotarget-08-52889-s002.doc]

**Supplementary Table 1:** **Clinico-pathological characteristics of 97 HNSCC of the first cohort and association with PD-L2 expression.**

|  | **Total number (n)** | **PD-L2 high** | **PD-L2 low** | **PD-L2 negative** | ***p* value** |
| --- | --- | --- | --- | --- | --- |
| **All HNSCC cases** | 97 | 2 (2.06%) | 81 (83.51%) | 14 (14.43%) |  |
|  |  |  |  |  |  |
| **Age** |  |  |  |  | ***p=0.367^†^*** |
| ≤50 years | 8 (8.25%) | 0 (0.00%) | 5 (62.50%) | 3 (37.50%) |  |
| 51-60 years | 27 (27.83%) | 1 (3.70%) | 23 (85.19%) | 3 (11.11%) |  |
| > 60 years | 62 (63.92%) | 1 (1.61%) | 53 (85.49%) | 8 (12.90%) |  |
| Median age [years] | 63 |  |  |  |  |
| Mean age [years] | 63.69 |  |  |  |  |
| Age range [years] | 38 - 84 |  |  |  |  |
|  |  |  |  |  |  |
| **Gender** |  |  |  |  | ***p=0.467^†^*** |
| Female | 16 (16.49%) | 0 (0.00%) | 15 (93.75%) | 1 (6.25%) |  |
| Male | 81 (83.51%) | 2 (2.47%) | 66 (81.48%) | 13 (16.05%) |  |
|  |  |  |  |  |  |
| **follow-up** |  |  |  |  |  |
| Mean follow-Up [days] | 566.66 |  |  |  |  |
| Median follow-Up [days] | 480 |  |  |  |  |
| Range follow-Up [days] | 4 -- 1814 |  |  |  |  |
|  |  |  |  |  |  |
| **Tobacco use** |  |  |  |  | ***p=0.159^†^*** |
| Non-smokers | 6 (6.18%) | 0 (%) | 4 (66.66%) | 2 (33.33%) |  |
| Smokers (current and former) | 40 (41.24%) | 1 (2.50%) | 36 (90.00%) | 3 (7.50%) |  |
| Unknown smoking status | 51 (52.58%) | 1 (1.96%) | 41 (80.39%) | 9 (17.65%) |  |
|  |  |  |  |  |  |
| **Alcohol consumption** |  |  |  |  | ***p=0.948^†^*** |
| No alcohol | 2 (2.06%) | 0 (0.00%) | 2 (100.00%) | 0 (0.00%) |  |
| Occasional | 5 (5.15%) | 0 (0.00%) | 4 (80.00%) | 1 (20.00%) |  |
| Moderate | 21 (21.65%) | 1 (4.76%) | 17 (80.95%) | 3 (14.29%) |  |
| Frequent | 7 (7.22%) | 0 (0.00%) | 6 (85.71%) | 1 (14.29%) |  |
| Alcoholic (current and former) | 7 (7.22%) | 0 (0.00%) | 7 (100.00%) | 0 (0.00%) |  |
| Unknown alcohol consumption | 55 (56.70%) | 1 (1.82%) | 45 (81.82%) | 9 (16.36%) |  |
|  |  |  |  |  |  |
| **HPV status** | No data available | | | | |
|  |  |  |  |  |  |
| **Localization** |  |  |  |  | ***p=0.823^†^*** |
| Oral cavity | 7 (7.22%) | 0 (0.00%) | 6 (85.71%) | 1 (14.29%) |  |
| Oropharnyx | 57 (58.76%) | 1 (1.76%) | 49 (85.96%) | 7 (12.28%) |  |
| hypopharnyx | 5 (5.15%) | 0 (0.00%) | 5 (100.00%) | 0 (0.00%) |  |
| larynx | 28 (28.87%) | 1 (3.57%) | 21 (75.00%) | 6 (21.43%) |  |
|  |  |  |  |  |  |
| **T-stage** |  |  |  |  | ***p=0.045^†^*** |
| Tis | 1 (1.03%) | 0 (0.00%) | 1 (100.00%) | 0 (0.00%) |  |
| T1 | 19 (19.59%) | 0 (0.00%) | 15 (78.95%) | 4 (21.05%) |  |
| T2 | 38 (39.18%) | 0 (0.00%) | 34 (89.47%) | 4 (10.53%) |  |
| T3 | 26 (26.80%) | 0 (0.00%) | 22 (84.62%) | 4 (15.38%) |  |
| T4 | 12 (12.37%) | 2 (16.67%) | 8 (66.66%) | 2 (16.67%) |  |
| Tx | 1 (1.03%) | 0 (0.00%) | 1 (100.00%) | 0 (0.00%) |  |
|  |  |  |  |  |  |
| **Lymph node involvement** |  |  |  |  | ***p=0.605^†^*** |
| N0 | 38 (39.18%) | 2 (5.26%) | 29 (76.32%) | 7 (18.42%) |  |
| N1 | 13 (13.40%) | 0 (0.00%) | 12 (92.30%) | 1 (7.70%) |  |
| N2 | 39(40.21%) | 0 (0.00%) | 34 (87.18%) | 5 (12.82%) |  |
| N3 | 2 (2.06%) | 0 (0.00%) | 2 (100.00%) | 0 (0.00%) |  |
| Nx | 5 (5.15%) | 0 (0.00%) | 4 (80.00%) | 1 (20.00%) |  |
|  |  |  |  |  |  |
| **Distant metastases** |  |  |  |  | ***p=0.893^†^*** |
| M0 | 65 (67.01%) | 2 (3.08%) | 53 (81.54%) | 10 (15.38%) |  |
| M1 | 1 (1.03%) | 0 (0.00%) | 1 (100.00%) | 0 (0.00%) |  |
| n.a. | 31 (31.96%) | 0 (0.00%) | 27 (87.10%) | 4 (12.90%) |  |
|  |  |  |  |  |  |
| **Grading** |  |  |  |  | ***p=0.005^†^*** |
| G1 | 2 (2.06%) | 0 (0.00%) | 0 (0.00%) | 2 (100.00%) |  |
| G2 | 53 (54.64%) | 0 (0.00%) | 47 (88.68%) | 6 (11.32%) |  |
| G3 | 39 (40.21%) | 2 (5.13%) | 31 (79.49%) | 6 (15.38%) |  |
| n/a | 3 (3.09%) | (0.00%) | 3(100.00%) | (0.00%) |  |
|  |  |  |  |  |  |
| **Lymphatic invasion** |  |  |  |  | ***p=0.851^†^*** |
| L0 | 67 (69.07%) | 1 (1.49%) | 57 (85.08%) | 9 (13.43%) |  |
| L1 | 21 (21.65%) | 0 (0.00%) | 18 (85.71%) | 3 (14.29%) |  |
| n/a | 9 (9.28%) | 1 (11.11%) | 6 (66.67%) | 2 (22.22%) |  |
|  |  |  |  |  |  |
| **Vascular invasion** |  |  |  |  | ***p=0.030^†^*** |
| V0 | 75 (77.32%) | 0 (0.00%) | 64 (85.33%) | 11 (14.67%) |  |
| V1 | 11 (11.34%) | 1 (9.09%) | 9 (81.82%) | 1 (9.09%) |  |
| n/a | 11 (11.34%) | 1 (9.09%) | 8 (72.73%) | 2 (18.18%) |  |
|  |  |  |  |  |  |
| **Extracapsular expansion** |  |  |  |  | ***p=0.219^†^*** |
| ece- | 21 (21.65%) | 0 (0.00%) | 15(71.43%) | 6 (28.57%) |  |
| ece+ | 28 (28.87%) | 0 (0.00%) | 24 (85.71%) | 4 (14.29%) |  |
| n/a | 48 (49.48%) | 2 (4.17%) | 42 (87.50%) | 4 (8.33%) |  |
|  |  |  |  |  |  |
| **Surgical margin** |  |  |  |  | ***p=0.800^†^*** |
| R0 | 69 (71.13%) | 1 (1.45%) | 56 (81.16%) | 12 (17.39%) |  |
| R1 | 19 (19.59%) | 1 (5.26%) | 16 (84.21%) | 2 (10.53%) |  |
| R2 | 1 (1.03%) | 0 (0.00%) | 1 (100.00%) | 0 (0.00%) |  |
| n/a | 8 (8.25%) | 0 (0.00%) | 8 (100.00%) | 0 (0.00%) |  |

†X^2^-test (Pearson)
