# Supplementary material for: PD-L1: a novel prognostic biomarker in head and neck squamous cell carcinoma
Source: Oncotarget. 2017 May 2;8(32):52889–900. doi: 10.18632/oncotarget.17547 (PMC5581079; doi:10.18632/oncotarget.17547)
Supplement: Supplementary file 3 [file oncotarget-08-52889-s003.doc]

**Supplementary Table 2: Clinico-pathological characteristics of 127 HNSCC of the second cohort and association with PD-L2 expression.**

|  | **Total number (n)** | **PD-L2 high** | **PD-L2 low** | **PD-L2 negative** | ***p* value** |
| --- | --- | --- | --- | --- | --- |
| **All HNSCC cases** | 127 | 13 (10.24%) | 108(85.04%) | 6 (4.72%) |  |
|  |  |  |  |  |  |
| **Age** |  |  |  |  | ***p=0.415^†^*** |
| ≤50 years | 17 (13.39%) | 1 (5.89%) | 16 (94.11%) | 0 (0.00%) |  |
| 51-60 years | 35 (27.56%) | 2 (5.71%) | 32 (91.43%) | 1 (2.86%) |  |
| > 60 years | 75 (59.05%) | 10 (13.33%) | 60 (80.00%) | 5 (6.67%) |  |
| Median age [years] | 63 |  |  |  |  |
| Mean age [years] | 62.95 |  |  |  |  |
| Age range [years] | 27 - 87 |  |  |  |  |
|  |  |  |  |  |  |
| **Gender** |  |  |  |  | ***p=0.706^†^*** |
| Female | 37 (29.13%) | 5 (13.51%) | 30 (81.08%) | 2 (5.41%) |  |
| Male | 90 (70.87%) | 8 (8.89%) | 78 (86.67%) | 4 (4.44%) |  |
|  |  |  |  |  |  |
| **follow-up** |  |  |  |  |  |
| Mean follow-Up [days] | 847.80 |  |  |  |  |
| Median follow-Up [days] | 763 |  |  |  |  |
| Range follow-Up [days] | 01.10.87 |  |  |  |  |
|  |  |  |  |  |  |
| **Tobacco use** |  |  |  |  | ***p=0.410^†^*** |
| Non-smokers | 12 (9.45%) | 0 (0.00%) | 12 (100.00%) | 0 (0.00%) |  |
| Smokers (current and former) | 76 (59.84%) | 6 (7.89%) | 66 (86.84%) | 4 (5.26%) |  |
| Unknown smoking status | 39 (30.71%) | 7 (17.95%) | 30 (76.92%) | 2 (5.13%) |  |
|  |  |  |  |  |  |
| **Alcohol consumption** |  |  |  |  | ***p=0.054^†^*** |
| No alcohol | 26 (20.47%) | 0 (0.00%) | 25 (96.15%) | 1 (3.85%) |  |
| Occasional | 20 (15.75%) | 4 (20.00%) | 16 (80.00%) | 0 (0.00%) |  |
| Alcoholic (current and former) | 36 (28.35%) | 1 (2.78%) | 34 (94.44%) | 1 (2.78%) |  |
| Unknown alcohol consumption | 45 (35.43%) | 8 (17.78%) | 33 (73.33%) | 4 (8.89%) |  |
|  |  |  |  |  |  |
| **HPV status** |  |  |  |  | ***p=0.812^†^*** |
| Negative | 115 (90.55%) | 12 (10.43%) | 98 (85.22%) | 5 (4.35%) |  |
| Positive | 12 (9.45%) | 1 (8.33%) | 10 (83.33%) | 1 (8.33%) |  |
|  |  |  |  |  |  |
| **Localization** |  |  |  |  | ***p=0.432^†^*** |
| Oral cavity | 43 (33.86%) | 6 (13.95%) | 34 (79.07%) | 3 (6.98%) |  |
| Oropharnyx | 50 (39.37%) | 3 (6.00%) | 46 (92.00%) | 1 (2.00%) |  |
| hypopharnyx | 14 (11.02%) | 1 (7.14%) | 13 (92.86%) | 0 (0.00%) |  |
| larynx | 20 (15.75%) | 3 (15.00%) | 15 (75.00%) | 2 (10.00%) |  |
|  |  |  |  |  |  |
| **T-stage** |  |  |  |  | ***p=0.589^†^*** |
| T1 | 28 (22.05%) | 1 (3.57%) | 26 (92.86%) | 1 (3.57%) |  |
| T2 | 47 (37.01%) | 6 (12.77%) | 39 (82.98%) | 2 (4.25%) |  |
| T3 | 31 (24.41%) | 4 (12.90%) | 24 (77.42%) | 3 (9.68%) |  |
| T4 | 18 (14.17%) | 2 (11.12%) | 16 (88.88%) | 0 (0.00%) |  |
| Tx | 3 (2.36%) | 0 (0.00%) | 3 (100.00%) | 0 (0.00%) |  |
|  |  |  |  |  |  |
| **Lymph node involvement** |  |  |  |  | ***p=0.767^†^*** |
| N0 | 51 (40.16%) | 3 (5.88%) | 45 (88.24%) | 3 (5.88%) |  |
| N1 | 23 (18.11%) | 3 (13.04%) | 19 (82.61%) | 1 (4.35%) |  |
| N2 | 44 (34.64%) | 7 (15.91%) | 36 (81.82%) | 1 (2.27%) |  |
| N3 | 1 (0.79%) | 0 (0.00%) | 1 (100.00%) | 0 (0.00%) |  |
| Nx | 8 (6.30%) | 0 (0.00%) | 7 (87.50%) | 1 (12.50%) |  |
|  |  |  |  |  |  |
| **Distant metastases** |  |  |  |  | ***p=0.758^†^*** |
| M0 | 121 (95.28%) | 12 (9.92%) | 103 (85.12%) | 6 (4.96%) |  |
| M1 | 6 (4.72%) | 1 (16.67%) | 5 (83.33%) | 0 (0.00%) |  |
| n.a. | 0 (0.00%) | 0 (0.00%) | 0 (0.00%) | 0 (0.00%) |  |
|  |  |  |  |  |  |
| **Grading** |  |  |  |  | ***p=0.847^†^*** |
| G1 | 4 (3.15%) | 1 (25.00%) | 3 (75.00%) | 0 (0.00%) |  |
| G2 | 68 (53.54%) | 6 (8.82%) | 59 (86.77%) | 3 (4.41%) |  |
| G3 | 33 (25.98%) | 3 (9.09%) | 29 (87.88%) | 1 (3.03%) |  |
| n/a | 22 (17.32%) | 3 (13.64%) | 17 (77.27%) | 2 (9.09%) |  |
|  |  |  |  |  |  |
| **Lymphatic invasion** |  |  |  |  | ***p=0.867^†^*** |
| L0 | 58 (45.67%) | 7 (12.07%) | 50 (86.21%) | 1 (1.72%) |  |
| L1 | 14 (11.02%) | 2 (14.29%) | 12 (85.71%) | 0 (0.00%) |  |
| n/a | 55 (43.31%) | 4 (7.27%) | 46 (83.64%) | 5 (9.09%) |  |
|  |  |  |  |  |  |
| **Vascular invasion** |  |  |  |  | ***p=0.044^†^*** |
| V0 | 63 (49.61%) | 5 (7.93%) | 57 (90.48%) | 1 (1.59%) |  |
| V1 | 8 (6.30%) | 3 (37.50%) | 5 (62.50%) | 0 (0.00%) |  |
| n/a | 56 (44.09%) | 5 (8.93%) | 46 (82.14%) | 5 (8.93%) |  |
|  |  |  |  |  |  |
| **Extracapsular expansion** |  |  |  |  | ***p=0.323^†^*** |
| ece- | 52 (40.94%) | 6 (11.54%) | 44 (84.61%) | 2 (3.85%) |  |
| ece+ | 16 (12.60%) | 4 (25.00%) | 12 (75.00%) | 0 (0.00%) |  |
| n/a | 59 (46.46%) | 3 (5.08%) | 52 (88.14%) | 4 (6.78%) |  |
|  |  |  |  |  |  |
| **Surgical margin** |  |  |  |  | ***p=0.044^†^*** |
| R0 | 90 (70.87%) | 11 (12.22%) | 77 (85.56%) | 2 (2.22%) |  |
| R1 | 9 (7.09%) | 0 (0.00%) | 7 (77.78%) | 2 (22.22%) |  |
| R2 | 2 (1.57%) | 0 (0.00%) | 2 (100.00%) | 0 (0.00%) |  |
| n/a | 26 (20.47%) | 2 (7.69%) | 22 (84.62%) | 2 (7.69%) |  |

†X^2^-test (Pearson)
